# Body weight gain during adulthood and uterine myomas: Pró-Saúde Study

**DOI:** 10.1590/S0034-8910.2015049005898

**Published:** 2015-11-10

**Authors:** Karine de Lima Sírio Boclin, Fernanda Pelegrini Torres, Eduardo Faerstein

**Affiliations:** Departamento de Epidemiologia. Instituto de Medicina Social. Universidade do Estado do Rio de Janeiro. Rio de Janeiro, RJ, Brasil

**Keywords:** Weight Gain, Leiomyoma, epidemiology, Risk Factors, Women’s Health

## Abstract

This study intended to investigate whether body weight gain during adulthood is associated with uterine myomas. 1,560 subjects were evaluated in a Pró-Saúde Study. Weight gain was evaluated in a continuous fashion and also in quintiles. Odds ratios and 95% confidence intervals were estimated through logistic regression models that were adjusted for education levels, color/race, body mass indices at age 20, age of menarche, parity, use of oral contraceptive methods, smoking, health insurance, and the Papanicolaou tests. No relevant differences were observed regarding the presence of uterine myomas among weight gain quintiles in that studied population.

## INTRODUCTION

Uterine myomas, also called leiomyomas or fibroid tumors, are benign tumors that develop slowly in several locations in the uterus. They are the most common tumors in the female reproductive system and, despite not being associated with increased risk of malignant tumors or mortality, significantly affect the quality of life of many women of reproductive age. Consequently, they are the main cause of hysterectomies in several countries.^2^

The origin and development of uterine myomas are believed to be influenced by the interaction between ovarian hormones, growth factors, cytokines, and components in the extracellular matrix.^2^ Thus, the most studied risk factors are the ones associated with the imbalance of circulating levels of estrogen and progesterone, such as high body mass index (BMI),^4^ higher percentage of fat,^3^ and weight gain during adulthood.^5^

Thus, exposure throughout or in the beginning of reproductive life are potentially crucial for the development of uterine myomas. Despite being frequently diagnosed in 40 to 50-year old women, its origin may date back to previous decades, as it is a tumor that develops slowly and frequently has no symptoms. In that sense, weight gain during adulthood may be a marker for circulating estrogen build-up throughout reproductive life.

The information on the relationship between weight gain and uterine myomas basically originated from two large American cohorts (Black Women’s Health and The Nurses’ Health Study);^1,4,5^ so far no Brazilian studies can be found on the topic. This study intended to test whether body weight gain during adulthood is associated with uterine myomas.

## METHODS

The Pró-Saúde Study is a longitudinal study that was conducted with technical and clerical workers from university campuses in Rio de Janeiro, RJ, Southeastern Brazil. It intended to evaluate the social determinants of health and health-related behaviors. Its baseline was conducted in 1999 and 2001, when 1,819 female workers (73.8% of the eligible ones) took part in data collection events. Data from 1,560 subjects of ages between 22 and 67 years were used. Eighty-six of those workers had been excluded for not having indications of uterine myoma, 73 for missing weight data at age 20 or in 1999, and 100 women were excluded for having lost weight since the age of 20.

Self-reported medical cases of uterine myoma were evaluated as dichotomous outcomes (yes;no) and collected from the following question: “Have you ever been informed by any physician you had a uterine myoma, or a benign uterine tumor?”.

Variable weight gain during adulthood, the main exposure, was built based on information regarding self-reported weight at age 20, through the following question: “How much did you use to approximately weigh (in kg) at the age of 20?”; and regarding weight in 1999. That information was included in the average weight gain/year calculation, considering the weight gain at age 20 and the collection of data in 1999, divided by the length of time between the age of 20 and the age in 1999. The variable was evaluated in a continuous fashion, and it was also categorized in quintiles with cutoff points of: 0.3; 0.5; 0.7; and 1.1 kg/year.

Different kinds of information were collected on the following variable categories: socioeconomic and demographic (color/race, education level), reproductive life (age of menarche, parity, and use of oral contraceptive methods), life style (smoking), and regarding access and use of health care services (health insurance, Papanicolaou and breast exams). They were analyzed as possible confounding variables.

The prevalences of self-reported medical diagnoses of uterine myoma were estimated in the whole population and in the subgroups that were determined by average weight gain per year quintiles, with respective 95% confidence intervals (95%CI). Odds ratios (OR) with 95%CI were estimated in the multivariate analyses through five logistic regression models: (Model 1) weight gain + BMI at age 20 + age; (Model 2) model 1 + socioeconomic and demographic variables; (Model 3) model 2 + reproductive life variables; (Model 4) model 3 + markers of access to health care; (Model 5) model 4 + smoking.

The data was entered and checked for consistency using the EpiInfo statistical package. The analyses were conducted in R statistical software, version 2.6.2.

The study was approved by the Research Ethics Committees of Hospital Universitário Pedro Ernesto (Records 224/1999 and 461/2001) and Instituto de Medicina Social (Record 005/2001). All subjects signed consent forms.

## RESULTS

Among the subjects in the study, 47.2% of them were young (ages between 35 and 44 years), 52.0% reported being white, 45.1% of subjects reported graduating from college, 73.1% had BMI between 18.5 and 24.9 kg/m^2^ at the age of 20, and 58.5% of the women reported having their menarches between ages 12 and 14. Most of them reported having one or two children (55.3%), health insurance (62.1%), having been submitted to Papanicolaou tests within the previous three years (88.6%), and having been submitted to clinical breast exams (88.2%).

In the average yearly weight gain quintiles, the prevalences were the following: 24.1% (95%CI 18.8;30.0) in the first one; 21.2% (95%CI 16.8;26.3) in the second one; 26.4% (95%CI 21.4;31.8) in the third one; 25.3% (95%CI 21.2;29.8) in the fourth one; and 19.1% (95%CI 14.9;23.9) in the fifth quintile (p = 0.174 – Pearson’s Chi-squared test).

The Table shows the multivariable analyses between average weight gain per year, self-reported medical diagnose of uterine myoma, and studied independent variables. The confidence intervals of all OR confirm the lack of bivariate association between uterine myoma and the average yearly weight gain quintiles. Even after the studied independent variables were included, both specific measures of association and the confidence intervals remained virtually unchanged, thus showing those variables did not influence the relationship between weight gain and uterine myoma.

**Table:** Prevalences, odds ratios (OR), and respective 95% confidence intervals in the association between average yearly weight gain and self-reported medical diagnose of uterine myoma. Pro-Health Study, Rio de Janeiro, RJ, Southeastern Brazil, 1999-2001.

Models	Weight gain^a^ (kg/year)		Weight gain quintiles (kg/year)	Prevalence		OR	95%CI
	
	OR	95%CI		n	%		
Crude model	0.8	0.6;1.0	1^st^ quintile (< 0.3)	57	24.1	1.0	
			2^nd^ quintile (≥ 0.3 < 0.5)	65	21.2	0.9	0.6;1.3
			3^rd^ quintile (≥ 0.5 < 0.7)	77	26.4	1.1	0.8;1.7
			4^th^ quintile (≥ 0.7 < 1.1)	104	25.3	1.1	0.7;1.6
			5^th^ quintile (≥ 1.1)	60	19.1	0.8	0.5;1.1
Model 1^b^	1.1	0.8;1.4	1^st^ quintile (< 0.3)	–		1.0	
			2^nd^ quintile (≥ 0.3 < 0.5)	–		0.8	0.6;1.3
			3^rd^ quintile (≥ 0.5 < 0.7)	–		1.1	0.7;1.6
			4^th^ quintile (≥ 0.7 < 1.1)	–		1.1	0.8;1.8
			5^th^ quintile (≥ 1.1)	–		1.0	0.7;1.6
Model 2^c^	1.1	0.9;1.4	1^st^ quintile (< 0.3)	–		1.0	
			2^nd^ quintile (≥ 0.3 < 0.5)	–		0.9	0.6;1.3
			3^rd^ quintile (≥ 0.5 < 0.7)	–		1.1	0.7;1.6
			4^th^ quintile (≥ 0.7 < 1.1)	–		1.2	0.8;1.8
			5^th^ quintile (≥ 1.1)	–		1.1	0.7;1.7
Model 3^d^	1.1	0.9;1.5	1^st^ quintile (< 0.3)	–		1.0	
			2^nd^ quintile (≥ 0.3 < 0.5)	–		0.9	0.6;1.4
			3^rd^ quintile (≥ 0.5 < 0.7)	–		1.1	0.7;1.8
			4^th^ quintile (≥ 0.7 < 1.1)	–		1.2	0.8;1.9
			5^th^ quintile (≥ 1.1)	–		1.2	0.7;1.9
Model 4^e^	1.1	0.8;1.4	1^st^ quintile (< 0.3)	–		1.0	
			2^nd^ quintile (≥ 0.3 < 0.5)	–		0.8	0.5;1.3
			3^rd^ quintile (≥ 0.5 < 0.7)	–		1.1	0.7;1.7
			4^th^ quintile (≥ 0.7 < 1.1)	–		1.1	0.7;1.7
			5^th^ quintile (≥ 1.1)	–		1.1	0.7;1.7
Model 5^f^	1.1	0.8;1.4	1^st^ quintile (< 0.3)	–		1.0	
			2^nd^ quintile (≥ 0.3 < 0.5)	–		0.8	0.5;1.2
			3^rd^ quintile (≥ 0.5 < 0.7)	–		1.1	0.7;1.7
			4^th^ quintile (≥ 0.7 < 1.1)	–		1.1	0.7;1.7
			5^th^ quintile (≥ 1.1)	–		1.1	0.7;1.7

^a^ Continuously analyzed variable.

^b^ Model 1: weight gain + BMI at age 20 + age.

^c^ Model 2: model 1 + socioeconomic and demographic variables.

^d^ Model 3: model 2 + reproductive life variables.

^e^ Model 4: model 3 + markers of access to health care.

^f^ Model 5: model 4 + smoking.

## DISCUSSION

No statistically significant differences were found, in the studied population, among the odds ratios for uterine myomas among weight gain quintiles. That result contradicts previous studies in North American populations, which reported increased risks among women who gained weight throughout adulthood.^1,4,5^

The several adjustments for socioeconomic and demographic, reproductive life, life style variables, and the ones regarding use of health care services have not changed the association found between weight gain and uterine myoma, which suggests those variables do not play confounding roles in that relationship. However, some limitations may have influenced such findings. The main one is related to a possible information error, as both the information on uterine myomas and the one on weight at the age of 20 depend on the subjects’ recollection. As uterine myomas are tumors that develop slowly and frequently have not symptoms, they may not have always been properly reported by women with restricted access to health care services and therefore to diagnoses.

Two strategies were used to minimize that limitation. The first one, an indirect one, was the test-retest reliability evaluation of the information on uterine myoma diagnosis, which was found to have an excellent standard (Kappa of 0.94 – 95%CI 0.86;1.00). The second one was the use of variables that were markers of access to health care services in the analyses; a large part of the studied population had reasonable conditions to get their tumors diagnosed – even if asymptomatic – as they had health insurance and had been submitted to Papanicolaou and breast tests less than three years before.

It should be said that the results might have been underestimated, due to the time elapsed between the treatment of uterine myoma and the weight gain, once women who have been diagnosed earlier might have stopped gaining weight during adulthood, as a result of their more careful attention to their bodies because of their tumors.

Our results may favor the hypothesis that the origin and development of these benign tumors may arise from hormonal environments and body compositions that are predominant in early adulthood (a period that precedes these analyses), even though their clinical signs and symptoms are mostly observed in pre-menopause age ranges.
