# Supplementary material for: Body weight gain during adulthood and uterine myomas: Pró-Saúde Study
Source: Rev Saude Publica. 2015 Nov 10;49:81. doi: 10.1590/S0034-8910.2015049005898 (PMC4634530; doi:10.1590/S0034-8910.2015049005898)
Supplement: Translation

# Translation: Ganho de peso corporal na vida adulta e ocorrência de miomas uterinos: Estudo Pró-Saúde

**Authors:** Karine de Lima Sírio Boclin, Fernanda Pelegrini Torres, Eduardo Faerstein

**Affiliations:** Departamento de Epidemiologia. Instituto de Medicina Social. Universidade do Estado do Rio de Janeiro. Rio de Janeiro, RJ, Brasil

**Keywords:** Ganho de Peso, Leiomioma, epidemiologia, Fatores de Risco, Saúde da Mulher

## Abstract

O objetivo deste estudo foi testar se ganho de peso na vida adulta está associado à ocorrência de miomas uterinos. Foram avaliadas 1.560 participantes do Estudo Pró-Saúde. O ganho de peso foi avaliado de forma contínua e em quintis. Razões de chances e intervalos de 95% de confiança foram estimados por modelos de regressão logística, com ajustes para escolaridade, cor/raça, índice de massa corporal aos 20 anos, idade da menarca, paridade, uso de contraceptivo oral, tabagismo, plano de saúde e exame Papanicolaou. Não foram observadas diferenças relevantes na ocorrência de miomas uterinos entre quintis de ganho de peso nessa população de estudo.

## INTRODUÇÃO

Os miomas uterinos, também chamados de leiomiomas ou tumores fibroides, são tumores benignos de evolução lenta que se desenvolvem em diversas localizações do útero. Constituem os tumores mais comuns do sistema reprodutor feminino e, apesar de não estarem associados ao aumento no risco de tumores malignos ou à mortalidade, afetam de forma significativa a qualidade de vida de muitas mulheres em idade reprodutiva. Como consequência, são a principal causa de histerectomia em diversos países.^2^

Acredita-se que origem e desenvolvimento dos miomas uterinos sejam influenciados pela interação entre hormônios ovarianos, fatores de crescimento, citocinas e componentes da matriz extra-celular.^2^ Assim, os fatores de risco mais estudados são aqueles associados à desregulação dos níveis circulantes de estrogênio e progesterona, como índice de massa corporal (IMC) elevado,^4^ maior percentual de gordura^3^ e aumento de peso na vida adulta.^5^

Dessa forma, exposições ocorridas ao longo da vida reprodutiva ou em seu início são potencialmente cruciais para o desenvolvimento de miomas uterinos. Apesar de frequentemente diagnosticados em mulheres entre 40 e 50 anos de idade, sua origem pode se dar em décadas anteriores, por se tratar de tumor de desenvolvimento lento e muitas vezes assintomático. Nesse sentido, o ganho de peso na vida adulta pode ser marcador do acúmulo de estrogênio circulante ocorrido ao longo da vida reprodutiva.

As informações sobre a relação entre ganho de peso e ocorrência dos miomas uterinos originam-se basicamente de duas grandes coortes norte-americanas (*Black Women’s Health* e *The Nurses’ Health Study*);^1,4,5^ e até o momento não foi possível identificar estudo brasileiro prévio sobre o tema. O objetivo deste estudo foi testar se ganho de peso na vida adulta está associado à ocorrência de miomas uterinos.

## MÉTODOS

O Estudo Pró-Saúde é uma pesquisa longitudinal realizada com trabalhadores técnico-administrativos de *campi* universitários localizados no Rio de Janeiro. Tem como foco avaliar os determinantes sociais da saúde e de comportamentos de saúde. Teve sua linha de base realizada nos anos de 1999 e 2001, quando participaram das coletas de dados 1.819 trabalhadoras (73,8% das elegíveis). Foram utilizados dados de 1.560 participantes com idade entre 22 e 67 anos, após exclusão de 86 sem informação sobre mioma uterino, 73 com dados faltantes sobre peso aos 20 anos, ou em 1999, e de 100 mulheres que perderam peso desde os 20 anos.

Casos de diagnóstico médico autorrelatados de mioma uterino foram avaliados como desfecho dicotômico (sim; não) e coletados a partir da questão: “Alguma vez um médico lhe informou que você tinha mioma uterino, um tumor benigno do útero?”.

A variável ganho de peso na vida adulta, exposição principal, foi construída com base nas informações do peso referido aos 20 anos, por meio da questão: “Aproximadamente, quanto você pesava aos 20 anos de idade? (kg)”; e do peso aferido em 1999. Essas informações foram inseridas no cálculo do ganho de peso médio/ano, considerado o ganho de peso entre os 20 anos e a coleta de dados em 1999 dividido pela duração do tempo entre os 20 anos e a idade em 1999. A variável foi avaliada de forma contínua e categorizada em quintis com pontos de corte de: 0,3; 0,5; 0,7; e 1,1 kg/ano.

Foram coletadas informações sobre variáveis socioeconômicas e demográficas (cor/raça; escolaridade), de vida reprodutiva (idade da menarca; paridade e uso de contraceptivo oral), de estilo de vida (tabagismo) e de acesso e utilização de serviços de saúde (plano de saúde; realização de exames Papanicolaou e de mama), sendo analisadas como possíveis variáveis de confusão.

Foram estimadas as prevalências com respectivos intervalos de 95% de confiança (IC95%) do diagnóstico médico autorrelatado de mioma uterino em toda a população e nos subgrupos determinados pelos quintis de ganho de peso médio ao ano. Nas análises multivariadas foram estimadas razões de chances (RC) com IC95% por meio de cinco modelos de regressão logística: (Modelo 1) ganho de peso + IMC aos 20 anos + idade; (Modelo 2) modelo 1 + variáveis socioeconômicas e demográficas; (Modelo 3) modelo 2 + variáveis da vida reprodutiva; (Modelo 4) modelo 3 + marcadores de acesso à saúde; (Modelo 5) modelo 4 + tabagismo.

A entrada dos dados e checagem de consistências foram realizadas com a utilização do pacote estatístico EpiInfo. As análises foram realizadas no programa estatístico R versão 2.6.2.

O estudo foi aprovado pelos Comitês de Ética em Pesquisa do Hospital Universitário Pedro Ernesto (Registros 224/1999 e 461/2001) e do Instituto de Medicina Social (Registro 005/2001). Todas as participantes assinaram termo de consentimento livre e esclarecido.

## RESULTADOS

Dentre as participantes do estudo, 47,2% eram jovens (idade entre 35 e 44 anos), 52,0% avaliaram sua cor/raça como branca, 45,1% tinham ensino universitário completo, 73,1% apresentavam IMC entre 18,5 e 24,9 kg/m^2^ aos 20 anos e 58,5%, idade da menarca entre 12 e 14 anos. A maioria, com um ou dois filhos (55,3%), relatou possuir plano de saúde (62,1%), ter realizado há menos de três anos o teste Papanicolaou (88,6%) e o exame clínico das mamas (88,2%).

Nos quintis de ganho de peso médio ao ano, as prevalências foram: 24,1% (IC95% 18,8;30,0) no primeiro; 21,2% (IC95% 16,8;26,3) no segundo; 26,4% (IC95% 21,4;31,8) no terceiro; 25,3% (IC95% 21,2;29,8) no quarto; e 19,1% (IC95% 14,9;23,9) no quinto quintil (p = 0,174 – Qui-quadrado de Pearson).

A Tabela apresenta as análises multivariáveis entre ganho de peso médio ao ano, diagnóstico médico autorrelatado de mioma uterino e as covariáveis estudadas. Os intervalos de confiança de todas as RC confirmam a análise bivariável de não associação entre mioma uterino e os quintis de ganho de peso médio anual. Mesmo após a inclusão das covariáveis estudadas, tanto as medidas de associações pontuais quanto os intervalos de confiança permaneceram praticamente inalterados, mostrando que não houve influência dessas variáveis na relação entre ganho de peso e mioma uterino.

**Tabela:** Prevalências e Razões de Chances (RC) e respectivos intervalos de 95% de confiança da associação entre ganho de peso médio anual e diagnóstico médico autorrelatado de mioma uterino. Estudo Pró-Saúde, Rio de Janeiro, RJ, Brasil, 1999-2001.

Modelos	Ganho de peso^a^ (kg/ano)		Quintis de ganho de peso (kg/ano)	Prevalência		RC	IC95%
	
	RC	IC95%		n	%		
Modelo bruto	0,8	0,6;1,0	1º quintil (< 0,3)	57	24,1	1,0	
			2º quintil (≥ 0,3 < 0,5)	65	21,2	0,9	0,6;1,3
			3º quintil (≥ 0,5 < 0,7)	77	26,4	1,1	0,8;1,7
			4º quintil (≥ 0,7 < 1,1)	104	25,3	1,1	0,7;1,6
			5º quintil (≥ 1,1)	60	19,1	0,8	0,5;1,1
Modelo 1^b^	1,1	0,8;1,4	1º quintil (< 0,3)	–		1,0	
			2º quintil (≥ 0,3 < 0,5)	–		0,8	0,6;1,3
			3º quintil (≥ 0,5 < 0,7)	–		1,1	0,7;1,6
			4º quintil (≥ 0,7 < 1,1)	–		1,1	0,8;1,8
			5º quintil (≥ 1,1)	–		1,0	0,7;1,6
Modelo 2^c^	1,1	0,9;1,4	1º quintil (< 0,3)	–		1,0	
			2º quintil (≥ 0,3 < 0,5)	–		0,9	0,6;1,3
			3º quintil (≥ 0,5 < 0,7)	–		1,1	0,7;1,6
			4º quintil (≥ 0,7 < 1,1)	–		1,2	0,8;1,8
			5º quintil (≥ 1,1)	–		1,1	0,7;1,7
Modelo 3^d^	1,1	0,9;1,5	1º quintil (< 0,3)	–		1,0	
			2º quintil (≥ 0,3 < 0,5)	–		0,9	0,6;1,4
			3º quintil (≥ 0,5 < 0,7)	–		1,1	0,7;1,8
			4º quintil (≥ 0,7 < 1,1)	–		1,2	0,8;1,9
			5º quintil (≥ 1,1)	–		1,2	0,7;1,9
Modelo 4^e^	1,1	0,8;1,4	1º quintil (< 0,3)	–		1,0	
			2º quintil (≥ 0,3 < 0,5)	–		0,8	0,5;1,3
			3º quintil (≥ 0,5 < 0,7)	–		1,1	0,7;1,7
			4º quintil (≥ 0,7 < 1,1)	–		1,1	0,7;1,7
			5º quintil (≥ 1,1)	–		1,1	0,7;1,7
Modelo 5^f^	1,1	0,8;1,4	1º quintil (< 0,3)	–		1,0	
			2º quintil (≥ 0,3 < 0,5)	–		0,8	0,5;1,2
			3º quintil (≥ 0,5 < 0,7)	–		1,1	0,7;1,7
			4º quintil (≥ 0,7 < 1,1)	–		1,1	0,7;1,7
			5º quintil (≥ 1,1)	–		1,1	0,7;1,7

^a^ Variável analisada de forma contínua.

^b^ Modelo 1: ganho de peso + IMC aos 20 anos + idade.

^c^ Modelo 2: modelo 1 + variáveis socioeconômicas e demográficas.

^d^ Modelo 3: modelo 2 + variáveis da vida reprodutiva.

^e^ Modelo 4: modelo 3 + marcadores de acesso a saúde.

^f^ Modelo 5: modelo 4 + tabagismo.

## DISCUSSÃO

Na população estudada, não foram encontradas diferenças estatisticamente significativas entre as razões de chances de ocorrência dos miomas uterinos entre os quintis de ganho de peso. Este resultado contrasta com estudos prévios em populações norte-americanas, os quais relataram aumento de risco entre mulheres que ganharam peso ao longo da vida adulta.^1,4,5^

Os diversos ajustes para variáveis socioeconômicas e demográficas, de vida reprodutiva, estilo de vida e de acesso e utilização de serviços de saúde não mudaram a associação encontrada entre ganho de peso e mioma uterino, sugerindo que estas variáveis não exercem confundimento nessa relação. No entanto, algumas limitações podem ter influenciado esses achados. A principal está relacionada ao possível erro de informação, visto que tanto a informação sobre os miomas uterinos quanto ao peso aos 20 anos são retrospectivas e dependem da memória das participantes. Como os miomas uterinos são tumores de evolução lenta e muitas vezes assintomáticos, seu relato pode estar subestimado entre mulheres com pouco acesso ao serviço de saúde e, consequentemente, ao diagnóstico.

Foram utilizadas duas estratégias para minimizar essa limitação. A primeira, indireta, foi a avaliação da confiabilidade teste-reteste da informação sobre diagnóstico de mioma uterino, que indicou padrão excelente (Kappa de 0,94 – IC95% 0,86;1,00). A segunda foi a utilização, nas análises, de variáveis marcadoras de acesso a serviços de saúde; grande parte da população de estudo teve condições razoáveis de diagnóstico médico dos tumores ainda que assintomáticos, por possuir plano de saúde e ter realizado teste Papanicolaou e exame de mama há menos de três anos.

Destaca-se a possibilidade de subestimação dos resultados decorrente do tempo ocorrido entre o tratamento do mioma uterino e o aumento de peso, uma vez que mulheres diagnosticadas há mais tempo podem ter interrompido o ganho de peso na vida adulta em decorrência do maior cuidado e atenção com o corpo por causa do tumor.

Nossos resultados podem favorecer a hipótese de que a origem e progressão desses tumores benignos sejam decorrentes do ambiente hormonal e da composição corporal predominantes no início da vida adulta (período que antecede as presentes análises), ainda que seus sinais e sintomas clínicos apareçam, em sua maioria, nas faixas etárias da pré-menopausa.
